# On “Ingrowing” of the Toe-Nail

**Published:** 1850-10

**Authors:** H. J. M’Dougal


### SURGERY.

*On "Ingrowing" of the Toe-Nail.* By H. J. M'Dougal, Esq.—[Mr. M'Dougal relates the following case: a gentleman was brought to him with an affection of the great toe which rendered him quite unable to walk, from the pain which pressure of the foot upon the ground occasioned. Mr. M'D. says:]

On examination, the toe was found slightly swollen, and with a reddish, erythematous blush extending up the foot. There was a very little fungoid granulation by the side of the nail, touching which was by no means so painful as pressure either on the under part of the toe, or on the upper and inner surface of the nail. The edge of the nail was quite invisible.

I directly proposed the usual operation of division of the nail in the centre, and eversion of the affected side. This had been proposed by two surgeons, whom the patient had previously visited, and was decidedly objected to. Being left to my own resources, therefore, I proceeded to scrape away, with an angle of glass, the inner surface of the nail (holding aside the flesh with the left hand) until its structure had become so thin that, with a pair of scissors, I was easily enabled to divide it for a short distance, and with forceps to lift out the piece in the corner. This gave little or no relief, and I was induced to seek further for the cause of the pain and distress felt on touching the toe. A horny-looking surface filled the space from which the piece of nail had been removed; and, on scraping round this with the point of the scissors, I succeeded in turning out a hardened mass of collected epithelium scales, nearly as large as the seed of a sweet pea. The surface underneath was red, and secreted a sanious matter. Perfect relief ensued on the removal of this extraneous matter, and the patient congratulated himself on his own obstinacy in not consenting to the very painful operation of losing half his nail. A morsel of dry lint completed his cure in twenty-four hours, and a little occasional attention to the part has since saved him from further suffering.

I am not aware that the condition I have described above has been noticed by any surgical writer in our language, with the exception of Mr. Colles, of Dublin, who refers to it as only occurring on one side of the nail. I can quite conceive, however, that with a little attention many persons might be saved the exquisitely painful and barbarous operation now so often used, of tearing asunder the inflamed matrix, confessedly one of the most tender parts in the whole structure of man.—*Med. Times, March* 16*th*, 1850, *p.* 195.

